# Circadian and Metabolic Perspectives in the Role Played by NADPH in Cancer

**DOI:** 10.3389/fendo.2018.00093

**Published:** 2018-03-15

**Authors:** Isabel Méndez, Mauricio Díaz-Muñoz

**Affiliations:** ^1^Departamento de Neurobiología Celular y Molecular, Instituto de Neurobiología, Universidad Nacional Autónoma de México, Querétaro, Mexico

**Keywords:** circadian, redox, NADPH, cancer, metabolism, Warburg effect

## Abstract

Physiological activity in healthy conditions requires a coordinated interaction between the molecular circadian clock and the network of biochemical pathways. An important metabolic parameter in the interface between these two entities is the redox state. Among the redox coenzymes that regulate the fluxes of enzymatic reactions is the NADP^+^/NADPH pair. Indeed, the main biosynthetic pathways need NADPH to serve as an electron donor for cellular anabolic transformations. The existence of a metabolic circadian clock is well established, and it was first identified in mammalian red blood cells. The metabolic circadian clock is independent of transcriptional activity and is sustained by the enzymatic complex peroxiredoxin/thioredoxin/NADPH. This complex shows 24-h redox fluctuations metabolizing H_2_O_2_ in various tissues and species (fungi, insects, and mammals). Although this NADPH-sensitive metabolic clock is autonomous in erythrocytes that lack a nucleus, it functions in concert with the transcriptional circadian clock in other cell types to accomplish the task of timing cellular physiology. During carcinogenesis, circadian alterations influence cell cycle onset and promote tumoral growth. These alterations also deregulate cellular energetics through a process known as aerobic glycolysis, or the Warburg effect. The Warburg effect is a typical response of cancer cells in which the metabolism turns into glycolysis even in the presence of functional mitochondria. This alteration has been interpreted as a cellular strategy to increase biomass during cancer, and one of its main factors is the availability of NADPH. This minireview explores the potential role of NADPH as a circadian and cancer-promoting metabolite.

## Metabolic and Transcriptional Clocks: Redox Homeostasis and Circadian Rhythms

The notion of divergent evolution in various timing systems in several model organisms is well established. For instance, cyanophytes, fungi, insects, and mammals show a set of clock genes that fluctuate daily but without a relationship between their DNA sequences. However, it was rapidly accepted that clock genes control circadian physiology through a network of positive and negative transcriptional loops [(1) and reference within]. Briefly, the positive elements in the primary feedback loop include CLOCK and BMAL1. CLOCK and BMAL1 heterodimerize and initiate transcription of target genes containing E-box *cis*-regulatory enhancer sequences, including PER and CRY. Negative feedback is achieved by PER:CRY heterodimers that translocate back to the nucleus to repress their own transcription by acting on the CLOCK:BMAL1 complex. Another regulatory loop is induced by CLOCK:BMAL1 heterodimers activating the transcription of retinoic acid-related orphan nuclear receptors, such as *Rev*-*erbα* and *Rorα*.

Nevertheless, many reports in the past 20 years question the robustness of the transcriptional circadian network as a unique form to sustain the biological measurement of time. It is known now that circadian oscillators are a complex system of transcriptional, posttranscriptional (phosphorylation, sumoylation, and acetylation), and metabolic integrated networks. Moreover, clock gene transcription is sensitive to the metabolic environment, which closely depends on the redox state. Interestingly, both nucleated and anucleated cells display self-sustained redox cycles that influence cellular physiology (2). Such is the case of suprachiasmatic neurons, whose excitability is guided by redox oscillation in a transcriptionally independent manner (2). In this regard, the existence of non-transcriptional rhythms was conclusively demonstrated by Dr. Akhilesh B. Reddy’s laboratory while studying human red blood cells (without nuclei in their mature form) (3). They reported the presence of a metabolic circadian oscillator based on the redox cycle of peroxiredoxin enzymes (4). Peroxiredoxins belong to a family of antioxidant enzymes whose main function is the catabolic degradation of hydrogen peroxide by controlling its levels and the associated signaling events (5). Peroxiredoxins are localized in several subcellular organelles. The catalytic mechanisms of peroxiredoxins involve the oxidation of a “reactive” cysteine residue in the active site to sulfenic acid (Cys-SOH), which then forms a disulfide bond with another non-catalytic cysteine residue. In some isoforms, such as 2-Cys peroxiredoxins, there is further oxidation to sulfonic and sulfonic acid forms. Eventually, the thioredoxin system reduces the disulfide bond using NADPH as a cofactor. These redox transformations have rapid turnover, resulting in low levels of intracellular hydrogen peroxide.

O’Neill and Reddy (3) reported circadian fluctuations of peroxiredoxin redox forms in erythrocytes, accompanied by daily variations of NADPH and NADH, and oxidized hemoglobin. Similar NADPH-dependent peroxiredoxin oscillatory systems have been detected in various organisms, including archaeal bacteria. Interestingly, such circadian rhythms are independent of canonical clock genes (6). At present, chronobiologists sustain that the metabolic/redox clock evolved following the Great Oxidation Event, when the Earth’s atmosphere became rich in oxygen. According to further evolutionary research, timing systems incorporated diverse circadian genes to reach the current timekeeping mechanism, which exhibits a dual modulation between the transcriptional circadian clock and the redox clock. The redox clock is represented by the NADPH-dependent peroxiredoxin oscillator and by the metabolic reactions mentioned in the first section of this review.

## NADPH as an Anabolic Coenzyme

Redox reactions involve a transfer of electrons (even naked or protonated electrons) between molecules. Thus, redox regulation of metabolism is carried out by conjugated redox pairs, with some molecules acting as donors (reducers) and others as acceptors (oxidizers). Thiols, such as glutathione (GSH, reduced form; GSSG, oxidized form), and coenzymes, such as flavin and nicotinamide adenine dinucleotides (FADH_2_/FAD^+^, FMNH_2_/FMN^+^, NADH/NAD^+^, and NADPH/NADP^+^), are key participants in metabolic regulation, as they modulate proteins that contain active sulfhydryl groups (enzymes, receptors, cytoskeletal proteins, among others), and the enzymatic activity of various dehydrogenases (7) and NAD^+^-dependent enzymes (8). Redox couples in an oxidation–reduction reaction are characterized by a standard redox potential ($E_{o}^{\prime }$, units in volts). $E_{o}^{\prime }$ is a measure of the affinity of a redox pair for electrons; negative values mean suitable electron donors, whereas positive values indicate adequate electron acceptors. The $E_{o}^{\prime }$ for redox coenzymes and glutathione is in the range of −0.32 V for NADH/NAD^+^ and NADPH/NADP^+^, −0.23 V for GSH/GSSG, and −0.22 V for FADH_2_/FAD^+^ and FMNH_2_/FMN^+^. The difference between the redox potentials of two redox pairs is a measure of the driving force for the net electron transfer and is related to the change in Gibbs free energy (Δ*G*). For example, Δ*G* under physiological conditions for the electron transfer from NAD(P)H to O_2_ is −52.6 kcal/mol (9). Under physiological conditions, the actual oxidation-reduction potential depends on the levels and ratio of the concentrations of the individual members of the redox couple, as well as on the prevalent pH. Each redox pair shows a defined ratio between its elements (reduced/oxidized) according to their subcellular compartments. The complete set of redox pairs makes up the global cellular redox state, a parameter that dictates the unique pattern of electron flux for any cell system.

In particular, the concentrations of NADP^+^ and NADPH within the cell are lower than those of NAD^+^ and NADH (submillimolar range), and under normal metabolic conditions the NADP pool is predominantly in its reduced form. NADPH primarily acts as an electron donor in anabolic or synthetic reactions (10). To accomplish this task, the pool is maintained in its reduced form (the NADPH/NADP^+^ ratio is kept high) (11). NADPH plays several biological roles [(12) and references within]. It is a coenzyme for glutathione reductase and transferase reactions, and it reactivates thioredoxin reductase and catalase as part of the antioxidant defense system. In addition, NADPH acts as an electron donor for the reductive formation of lipid molecules (cholesterol and fatty acids) and nucleic acids. It is a cofactor for $O_{2}^{-}$ generation during NADPH oxidase activity and a protector of mitochondrial DNA integrity. NADPH also acts as a nuclear modulator of gene expression by promoting redox signaling within the nucleus. Finally, it has been shown that NADPH, as a product of the pentose phosphate pathway (PPP), is able to modulate circadian rhythms by extending or shortening the 24-h fluctuations in human cells, mouse tissues, and fruit flies (13).

## Circadian Disruption and Cancer

Misalignment of the circadian clock with the environment seems to lead to various health alterations such as metabolic diseases and some types of cancer (14). In humans, epidemiological evidence of the adverse association between shift work and disrupted sleep/wake schedules in healthy conditions supports that knowledge (15). In animal models, disruption of circadian cycles by exposure to light at night dramatically accelerates tumorigenesis and tumor growth (16, 17). In fact, entrainment by restricted feeding inhibits tumor growth in mice with pancreatic adenocarcinoma, with no alteration of the arrhythmic clock gene expression in the tumor in contrast to the synchronization effect in the liver, irrespective to calorie intake. However, genes involved in cell cycle and metabolism were upregulated or downregulated, depending on the circadian time (18). This evidence supports the fact that, aside from the transcriptional regulation of circadian time, other levels of regulation are implicated in the alteration of healthy homeostasis. The fact that resynchronization by restricted feeding delays tumor development highlights the role of metabolism and redox status in tumor growth and progression (19).

Recent studies have shown that peroxiredoxins contribute significantly to the promotion and progression of cancer. Members of the peroxiredoxin family are seemingly overexpressed in several tumor tissues (20–22), and they promote cell proliferation and tumorigenesis through epithelial–mesenchymal transition (23, 24). Hyperoxidation of peroxiredoxins by hydrogen peroxide induces their inactivation, and the sulfiredoxin reductive action reactivates them. In fact, a hyperoxidized form of peroxiredoxin III and sulfiredoxin is in antiphase circadian oscillation in healthy cells (25). Overexpression of peroxiredoxins with a decrease in sulfiredoxin in some neoplasias correlates with poor prognosis (26). However, it is not known if overexpression of peroxiredoxins is due to changes in a rhythmic profile of activation by NADPH that contribute to tumor development in a protective redox role of peroxiredoxins.

On the other hand, Myc family oncoproteins (c-Myc, N-Myc, and L-Myc) regulate the transcription of several genes, some of them implicated in the shuttling of glucose to activate the PPP, resulting in the generation of large amounts of NADPH and the biosynthesis of various macromolecules (27). In addition, it has been demonstrated that Myc disrupts the circadian molecular clock. Specifically, it activates the Bmal1–Clock heterodimer, thus disrupting circadian metabolic oscillation (28). This effect occurs through constitutive activation of Rev-erbα, the expression of which could be related to poor clinical outcome in human neuroblastoma (28). These observations highlight the interplay between redox state and circadian clock in cancerous processes.

## Toward a Circadian Characterization of the Warburg Effect?

The onset and development of carcinogenic growth involves a multistep process characterized by a set of biological features known as hallmarks of cancer. The initial list of hallmarks encompassed characteristics such as replicative immortality, angiogenesis, and metastasis (29). Recently, new cellular and biochemical parameters were incorporated into the list of hallmarks (30). One of them, the cancer-associated reprogramming of energy metabolism, also known as the Warburg effect, is characterized by predominant glycolytic activity despite aerobic conditions and functional mitochondria. In cancer, the Warburg effect is related to an upregulation of WNT/β-catenin signaling and a concomitant downregulation of the PPARγ-associated actions (31). The rationale of the Warburg effect in oncology is that cancerous cells are programmed for high cellular proliferation; hence, the continuous entry into the cell cycle involves a constant input of new molecules for the synthesis of biological membranes, genetic material, and all the cellular elements needed for the newly formed tumoral cells (32). This anabolic commitment is fulfilled by an upgraded availability of NADPH during neoplastic growth, since this coenzyme is required by the reductive biosynthetic reactions of a duplicating cell (33). NADPH can be generated by the activities of (1) glucose-6-phosphate dehydrogenase and 6-gluconate phosphate dehydrogenase (redox and decarboxylating steps in the PPP); (2) NADP^+^-dependent isocitrate dehydrogenase (mitochondrial isozyme that provides NADPH for antioxidant activity); (3) NADP^+^-dependent malic enzymes (important cytosolic enzymes during the β-reduction reactions); and (4) transhydrogenase (mitochondrial enzyme that is also part of the antioxidant defense mechanisms). Along with higher NADPH formation, the anabolic response associated with the Warburg effect requires the enzymatic activity of citrate lyase, which provides carbon skeletons for fatty acid synthesis (12).

The relationship between circadian rhythms and the Warburg effect has been scarcely explored (from 1,911 entries in PubMed in December 2017 with the keyword “Warburg effect,” only 9 are related to circadian rhythms). For example, in 2017, Cao and Wang reported on the potential connection between circadian responses, providing examples of metabolic reprogramming and offering interesting insights into the onset and development of tumors (34). Four other reports specifically explored the effect of light exposure and the concomitant increase in melatonin as cancer suppressors by disrupting the Warburg effect in human breast and prostate cancer xenografts, as well as in leiomyosarcoma (35–37). Dr. Vallee’s group has made interesting thermodynamic considerations regarding the equilibrium between WNT/β-catenin and PPARγ signaling in fibrosis and glioma; the first favors the Warburg effect by reprogramming cellular energy metabolism, and the second promotes a reduction in circadian physiology upon its inactivation (31, 38, 39).

A more formal approach to analyze the importance of 24-h fluctuations in the Warburg effect and the availability of NADPH in cancer needs to consider that redox influence of NADPH and NADH is completely dissimilar (40). Given that the former is a coenzyme for anabolic reactions and the latter for catabolic reactions, the metabolic context for the action of each one is necessarily different; hence, reports that refer to both coenzymes as NAD(P)H and claim a regulatory redox event are conceptually mistaken. Another common mistake is to consider that the cellular redox state can be inferred from the synthesis, presence, or activity of nicotinamide phosphoribosyltransferase. This enzyme allows the formation of nicotinamide adenine dinucleotides; however, the redox state is necessarily defined by the ratio of the redox couple (reduced/oxidized) (40).

Indirectly, the reduced role of PPARγ in cancerous cells (31) could be associated with the damped circadian rhythms mentioned in the previous section. Specifically, there are few reports on circadian regulation of the enzymes responsible for NADPH availability during the Warburg effect, and almost none regarding their daily rhythms in cancerous cells or tumors. As an exception, the activity of the PPP, one of the major generators of NADPH, has been recognized as an element of circadian physiology in various cell systems (13).

## Summary

**Figure 1:**
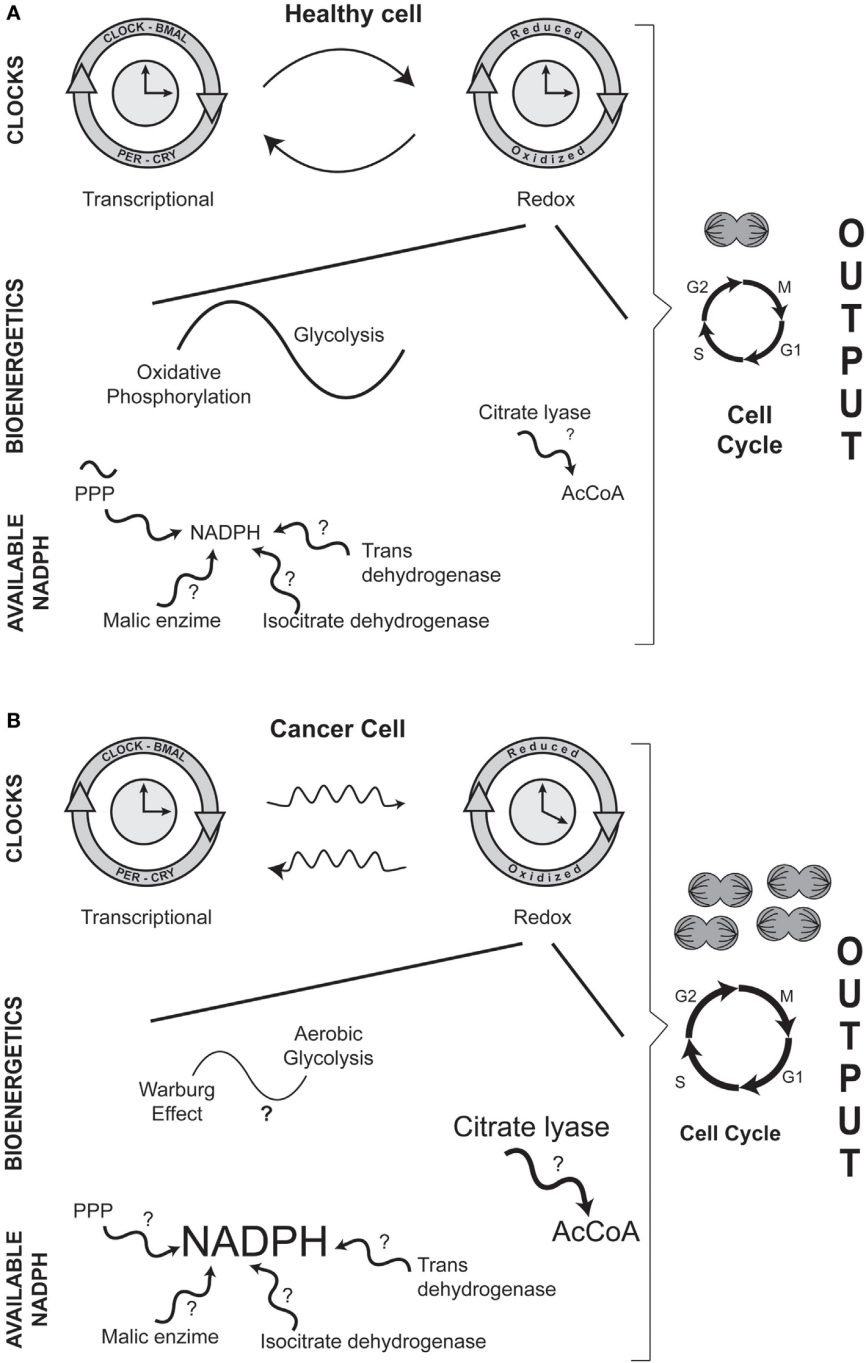
Comparison between healthy **(A)** and cancer **(B)** cells: circadian and metabolic differences in NADPH cellular handling. In both entities, there is a dual interaction between the transcriptional and the redox circadian clocks. However, in cancerous cells, the daily variations in biochemical and molecular phenomena are disrupted. A major difference between a normal and a transformed cancer cell is the bioenergetic adaptation in which the main mitochondrial activity is no longer ATP production (glycolysis/oxidative phosphorylation en healthy cells) but the constant input of metabolic intermediaries (Warburg effect, or aerobic glycolysis, in cancerous cells) that are needed for cellular duplication. A distinctive biochemical characteristic in carcinogenesis is the increased availability of the anabolic coenzyme, NADPH; NADPH can be potentially be formed by various enzymatic reactions. Regarding the higher levels of NADPH, the production of acetyl-CoA (AcCoA) is also increased by the activity of the cytoplasmic enzyme citrate lyase. In the figure, events or enzymatic reactions that show circadian rhythmicity are depicted by the symbol ~; for example, circadian rhythmicity of the pentose phosphate pathway (PPP) in normal cells. Question marks (**?**) indicate events or enzymatic reactions that have not been characterized as showing putative daily rhythmicity in either healthy or cancer cells.

The conceptual message of this minireview is outlined in Figure 1. (1) Carcinogenesis involves altered circadian physiology and a modified relation between transcriptional and redox clocks; (2) the main role of mitochondria is no longer ATP synthesis; (3) bioenergetic is transformed from glycolysis/oxidative phosphorylation into aerobic glycolysis (Warburg effect); (4) the metabolic networks are oriented toward anabolic reactions; (5) NADPH is more available to ensure biosynthetic reactions; (6) cellular replicative function is enhanced.

Although daily variations in redox mechanisms are well established in healthy cells, some reactions associated with NADPH metabolism have not been well characterized in terms of 24-h rhythmicity (question marks in Figure 1). The situation is even more accentuated in cancerous cells, since few reports have approached the onset and development of NADPH availability and the Warburg effect from a circadian perspective. Undoubtedly, the characterization of circadian rhythmicity of NADPH formation in healthy and neoplastic cells, as well as the Warburg effect in cancer, will be promising fields of opportunity for laboratories interested in studying redox adaptations in the physiopathology of the circadian timing system.

## Author Contributions

MD-M and IM participated in the conceptual aspect of the manuscript, wrote several sections of the minireview, and agreed with the final version of the manuscript.

## Conflict of Interest Statement

The authors declare that the research was conducted in the absence of any commercial or financial relationships that could be construed as a potential conflict of interest.
